# Glioblastoma Stem Cells: A Neuropathologist's View

**DOI:** 10.1155/2011/397195

**Published:** 2010-11-01

**Authors:** Roger E. McLendon, Jeremy N. Rich

**Affiliations:** ^1^Department of Pathology, Duke University Medical Center, Durham, NC 27710, USA; ^2^Department of Stem Cell Biology and Regenerative Medicine, Lerner Research Institute, Cleveland Clinic, Cleveland, OH 44195, USA

## Abstract

Glioblastoma (WHO Grade IV) is both the most common primary brain tumor and the most malignant. Advances in the understanding of the biology of the tumor are needed in order to obtain a clearer picture of the mechanisms driving these tumors. To neuropathologists, glioblastoma is a tumor that represents a complex system of migrating pleomorphic tumor cells, proliferating blood vessels, infiltrating inflammatory cells, and necrosis. This review will highlight how the glioma stem cell concept brings these elements together into a collective whole, interacting with microenvironmental influences in complex ways. Borrowing from chaos theory a vocabulary of “self organizing systems” and “complex adaptive systems” that seem useful in describing these pathologic features, a new paradigm of glioblastoma biology will be proposed that genetic changes should be understood in a three dimensional framework as they relate not only to the tumor cells themselves but also to the multicellular hierarchical unit, not isolated from, but responsive to, its local milieu. In this way we will come to better appreciate the impact our therapeutic interventions have on the regional phenotypic heterogeneity that exists within the tumor and the intercellular communications directing adaptation and progression.

Glioblastoma (WHO Grade IV) is both the most common primary brain tumor and the most malignant. Advances in the understanding of the biology of the tumor are needed in order to obtain a clearer picture of the mechanisms driving these tumors. The advances obtained to date have challenged those of us who work with glioblastoma on a daily basis to keep opposing ideas in our minds. For example, while glioblastoma is a uniform diagnostic category of astrocytic tumors, the intertumoral and intratumoral histologic heterogeneity within them reflects a chaotic diversity of cell types and mesenchymal backgrounds. Also, while molecular data indicate that these tumors have a hypermutable genotype [1] and many different cytologic repertoires [2, 3] available to them, recurrences often exhibit histologic features similar to the primary tumors (personal observation). What is driving the microenvironmental organization of necrosis and microvascular proliferation that these tumors recapitulate so robustly as to represent the key diagnostic features [4] of the tumor, however, is not clear. The recent identification of malignant cells within glioblastomas with stem-cell-like qualities provides insights into these questions. Furthermore the recent advances in chaos theory have provided a vocabulary of “self organizing systems” and “complex adaptive systems” that seem useful in describing these pathologic features. In order to better understand where we are now, it is useful to review some early conceptual issues related to grading malignant astrocytomas.

In the 1920s, Bailey and Cushing proposed a cytogenetic paradigm of glioblastoma classification in which a relationship between gliomas and undifferentiated cells in glioblastomas was hypothesized [5]. This immediately fell under attack as the embryologic systems had no equivalent of a glioblast. Indeed, a major problem of the cytogenetic system was that it classified glioblastomas as *de novo* tumors, ignoring elements of intermediate differentiation and malignant progression from lower grade tumors. Subsequently, malignant progression was approached by Kernohan [6], Ringertz [7], and Earle and colleagues [8] in a variety of ways. The major problem inherent in the cytogenetic approach lay not only in its neglect of the nonglial features of the tumors including angiogenesis, mesenchymal elements, and even necrotic foci that have become landmarks of grading the tumor [4, 9] but also they all emphasize a linearity in progression from early tumor to later tumor that are not found in nature. Rather, the appearance of glioblastoma histology is one of explosive growth with a rapid appearance of mesenchymal features not evident in lower grade tumors. The recent description of glioma cells with stem cell qualities and mesenchymal interactions provides a nidus of organization around which accelerated growth, therapeutic resistance, and mesenchymal proliferation appear to be centered. As such, the glioma stem cells provide a potential unifying concept with which to better understand the histologic appearances of these tumors.

The concept of a malignant cell with stem cell qualities arose very early in the history of anatomic pathology as Virchow, himself, noted the similarities between certain cancers and embryogenetic processes and ascribed the origins of cancers to embryonic rests (for which proof resides in certain pediatric brain tumors [10]). Subsequently, other researchers found evidence to support the presence of a rare cell in tumors capable of regenerating the tumors and forming colonies in cell culture (reviewed in [11]). However, the origin of these purported tumor stem cells is not at all clear. In contrast to embryogenesis where the normal stem cell has a known origin from primitive precursors, the tumor stem cell origin from stem cells versus transient amplifying cells derived from stem cells versus dedifferentiated mature cells is far from being solved.

The possibility of brain tumor stem cells arose from the concepts proposed in 1982 by ML Rosenblum and colleagues who hypothesized inherent differences in the sensitivity of clonogenic cells as an explanation for clinical drug failure, tumor heterogeneity, and age-response relationships [12]. This was followed by the demonstration of complex heterogeneity in the human glioma cell line, D54, and eight-derived clones by Wikstrand and colleagues establishing the intrinsic biologic variation that seemed to underlie the multiagent resistance of glioblastoma [13]. Singh and colleagues in the Dirks laboratory [14], working on pediatric brain tumors, were also struck by the heterogeneity of these tumors with respect to proliferation and differentiation and their similarity to human leukemia. In leukemias, tumor clones exhibit hierarchical organization originating from rare leukemic stem cells that possess extensive proliferative and self-renewal potential and are responsible for maintaining the tumor clone [15, 16]. Similar cells with stem-like qualities were subsequently confirmed in the solid tumor systems, glioma [17] and adenocarcinoma of the breast [18]. Singh and colleagues used the cell surface marker CD133 (prominin-1) to sort out a clonogenic population of cells demonstrating stem-like features in medulloblastomas and pilocytic astrocytomas with capabilities of self-renewal and multilineage differentiation and declared these cells to be brain tumor stem cells [14]. Several groups later confirmed these findings in other brain tumors, including glioblastomas [19, 20]), medulloblastomas [14, 21, 22], and low grade gliomas [14], and ependymomas [23] also display functional heterogeneity with a potential hierarchy of differentiation like that noted in a stem cell system.

The tumor stem cell hypothesis adds to the conventional cytogenetic theory in ways that are both complementary and explanatory. The conventional approach hypothesizes a probabilistic approach to clonal emergence within a tumor mass whereby a “lucky” cell happens to exhibit those properties necessary to survive, recur, or spread within its microenvironmental setting. It implies that the “lucky” cells are not necessarily similar in any way; chance places a cell with the correct combination of abilities in the right place to survive and progress. However, research into the glioma stem cells described above has revealed attributes within a minor population of tumor cells that have significantly altered our thinking. These findings indicate that while the masses of tumor cells are clonal and share a common cytogenesis, there is a hierarchical growth pattern within the tumor with cells capable of adaptive responses to the microenvironment in a way that not only maintains the cellular heterogeneity of the tumor but also uses this heterogeneity to manifest emergent behaviors of tumor progression. These rare cells exhibit features of a central organizer. The role of organizing and driving tumoral adaptation in glioblastomas occurs in a number of ways, such as supporting growth, promoting heterogeneity, endowing therapeutic resistance, and remodeling the mesenchymal and microvascular environment (Figure 1).

In 2006, Bao and colleagues [24], using the CD133 antibody, selected cells from glioblastoma biopsies that were not only capable of forming tumorspheres in culture but also demonstrated self-renewal and multilineage differentiation. An immunohistochemical interrogation of these CD133+ cells further revealed the coexpression of a number of markers commonly associated with benign stem cells including SOX2, Musashi, and Nestin. The tumorspheres, when transplanted into nude mouse intracranial models, were capable of forming tumors recapitulating the glioblastoma phenotype. In contrast, the CD133 negative (CD133−) cells could neither form spheroids in normoxic conditions nor form tumors when xenografted. However, when cocultured with CD133+ cells, the CD133− cells formed tumors, the xenograft implantation rate of which was dose dependent upon the percentage of cocultured CD133+ cells. When biopsies of glioblastomas were investigated, CD133+ cells proved to be a minor population of cells varying from 1 to 3% of total tumor cell population; however, clinical studies suggested that the percentage of CD133+ cells [25, 26] or rate of tumorsphere formation [27, 28] predicted overall survival of the patient (although contrary evidence also exists [29]).

In their initial paper, Bao and colleagues found that CD133+ cells constitutively activated the DNA repair genes CHK1 and CHK2 at much higher levels than CD133− cells, and this expression mediated resistance to X-irradiation. When the checkpoint response enzymes were specifically inhibited, the CD133+ cells became as susceptible to radiation as the CD133− cells [24].

The CD133+ cells were also found to exhibit features indicative of an abrogation of programmed cell death pathways. Programmed cell death is necessary for the maintenance of a normal healthy cellular population and is frequently implicated in cell death due to chemotherapeutic interventions. For survival, tumors must overcome natural self-destruction signals generated by these internal and external signals. In normal development, MYC drives the undifferentiated states of developing progenitor cells by combining with Max, a prodifferentiation agent. Gradually, Mad proteins displace Myc from the MYC-Mad duplex allowing the formation of Max-Mad complexes which elicit differentiation inducing signals. However, recent studies indicate that MYC proteins are overexpressed in glioma stem cells and drive not only an undifferentiated state but also trigger an antiapoptotic, prolife signaling cascade in the cells [30–32]. A prominent upstream factor stabilizing MYC complexes is the HIF2*α* pathway, an important pathway related to hypoxia that is discussed in more detail below [33].

Another major regulator of cell survival in GSCs is tumor necrosis factor alpha-induced protein 3 (TNFAIP3), or A20. Hjelmeland [34] determined that A20 is overexpressed in glioma stem cells relative to nonstem glioblastoma cells. Elevated levels of A20 in glioma stem cells contribute to apoptotic resistance via loss of susceptibility to TNF*α*-induced cell death. A20 knockdown sensitized GSCs to TNF*α*-mediated apoptosis as well as decreased GSC survival, self-renewal, and tumor growth. These findings contrast to lymphomas in which loss of A20 via mutations suggests that A20 acts as a tumor suppressor. These data suggest that A20 may function as a tumor enhancer in glioma through promotion of glioma stem cell survival.

In like kind to benign neural stem cells [35], the organizational significance of rare glioblastoma cells with stem-cell-like qualities extends far beyond the confines of the cell's cytoplasmic borders. Rather, the major organizational and adaptational impact of CD133+ cells lies in their influence on the cells and mesenchymal elements about them. Recent studies have revealed a potential amplifying feedback mechanism to be extant between CD133+ and CD133− cells involving the progrowth factor, Interleukin 6 (IL6). Recent studies by Wang and colleagues [36] indicate that the CD133− cells express IL6 but express few, if any, receptor components on their cell surface. However, the CD133+ cells express the components of IL6 receptor, gp160 and IL6 receptor alpha, on their cell surface while making less IL6 itself. The significance of IL6 lies in the fact that its signals promote STAT3 activation in GBM cells in vitro, and targeting of either STAT3 or IL6 decreases GBM cell survival [37–40] and appears to promote invasion [41]. Additional reports also link STAT3 to stem cell biology as STAT3 is required to maintain the propagation and pluripotency of normal embryonic stem cells, neural stem cells [42–44], and glioblastoma stem cells [40, 45]. Also STAT3 has been linked to mesenchymal differentiation in glioblastoma cells, a phenotype of clinical aggressiveness and poor survival [46]. Furthermore, clinically IL6 is important as the quantitative load of IL6 and its receptors also correlate inversely with patient survival [47].

The extra-cellular influence of CD133+ cells is also manifest on the stromal cells about them. Histologically, brain tumor stem cells seem to prefer a perivascular niche [48–50], a location that recapitulates the normal neural stem cells and the vasculature of the developing central nervous system [35, 51]. Glioma stem cells have been shown to mediate vascular proliferation in glioblastomas via Vascular Endothelial Growth Factor (VEGF). VEGF is chemically activated via the Hypoxia Inducible Factor (HIF) pathway. In glioblastomas, HIF1*α* is found in all malignant cells, while HIF2*α* expression is unique to glioma stem cells. Under mild hypoxic conditions, HIF2*α* is stabilized preferentially while HIF is not present, leaving HIF2*α* as the active primary signaling agent driving the downstream expression of VEGF. However, the hierarchical interplay between glioma stem cells and vessels is not for nutritional needs alone as the glioma stem cells have also been found to express the laminin receptor, integrin *α*6.

This integrin is a key receptor for laminins found in the extracellular matrix (ECM) of vessels. Integrin *α*6 is produced in the glioma stem cells and is found to mediate growth and maintenance of glioma stem cells by knockdown studies. This is significant in that the extracellular matrix surrounding normal neural stem cells is known to provide both structural and instructive cues [52] within the CNS. Several reports have suggested that ECM structures originating from blood vessels in the adult neural stem cell germinal zones are critical in preserving their maintenance through serving as a reservoir for growth factors [53]. Thus, the potentiality of a perivascular niche rich in oncogenic signals further highlights the interplay at work among glioma stem cells, malignant nonstem glioma cells, and the mesenchymal elements earlier appreciated by Rubinstein and other pathologists. Indeed, the findings strengthen the declaration that tumors are aberrant organ systems that display a complex organizational interplay among neoplastic cells, recruited vascular, inflammatory, and stromal elements [54].

The perivascular niche is also important to stem cell physiology. Oxygen tension is tightly regulated in normal development with low oxygen tension associated with maintenance of an undifferentiated state. Hypoxia promotes the self-renewal of embryonic stem cells and prevents the differentiation of neural stem cells *in vivo*  [55–57]. Hypoxia is also likely to be a functional component of a normal stem cell niche as well. Hematopoietic stem cells are maintained in bone marrow within hypoxic niches [58]. In regards to this, hypoxia has been shown to increase expression of stem cell markers in neuroblastomas, erythroleukemia, and cell lines [59–61]. These findings suggested to Li and colleagues [62] that the hypoxic environment of a glioblastoma may promote stem cell survival. Indeed, they found that targeting HIFs in glioma stem cells inhibited self-renewal, proliferation, and survival in vitro and attenuated tumor initiation potential of these cells *in vivo*. They found, as well, that HIF2*α* expression correlated with poor patient survival. Heddleston and colleagues [33] followed with data indicating that hypoxia promoted the self-renewal capability of the stem and nonstem population as well as promoted a more stem-like phenotype in the nonstem population. This was corroborated by increased tumorsphere formation as well as upregulation of important stem cell factors, such as OCT4, NANOG, and MYC. The importance of HIF2*α* was also supported via experiments demonstrating that forced expression of nondegradable HIF2*α* induced a cancer stem cell phenotype as well as augmented the tumorigenic potential of the nonstem population. Furthermore, HIF2*α* is known to mediate changes associated with clinically aggressive behavior including dissemination as well as angiogenesis. This susceptibility of the nonstem glioma population to transition to a stem-like phenotype emphasizes the importance of microenvironmental influences on the tumor and the remarkable capacity within glioma cells to adapt to hypoxia.

The multiple potentialities of a subpopulation of glioblastoma cells for self-maintenance, multilineage differentiation, and self-renewal identify them as having stem cell like qualities. However, the complex interactions among stem cells, nonstem cells, and mesenchymal cells mediate the cytologic heterogeneity well recognized by neuropathologists for decades. The presence of cells with embryonal features further lends support to these features identified by the earliest neuropathologists. The presence of vascular proliferation as a required component for grading tumors marks astrocytoma as the only tumor system in which the vascular component is a requisite feature for characterizing prognosis. Details identified by glioma stem cell researchers have characterized a more complex interaction than was previously appreciated.

Modern research makes clear that glioblastomas do not behave as an organic whole; geographic heterogeneity arises as the tumor regionally adapts to the microenvironment the individual cell clusters find themselves in (Figure 2). For example, recent studies have indicated that antiangiogenic therapy results in short-term tumor burden control but does not affect overall survival. Rather, escape mechanisms include some tumors responding with migration, producing the gliomatosis cerebri state (19567589) or responding with increases in basic Fibroblast Growth Factor (bFGF), Stromal cell-derived factor 1*α* (SDF1*α*), and viable circulating endothelial cells (CECs) [63]. In this regard, the tumor behaves as a complex adaptive system [64] exhibiting emergent behavior. While the main body of tumor is relatively homogeneous with respect to genotype (driving genetic mutations) [65], it is heterogeneous with respect to phenotypic diversity; the offspring of glioma cells not only diverge and differentiate cytologically, but the stem-cell offspring are also driven to seek viable options. Furthermore, evidence suggests that remnant clones of earlier stages remain as reserve cells, capable of generating tumoral renewal. Adaptational forces resulting from microenvironmental influences of hypoxia, vascularity, acidic stress, starvation, exogenous growth factors, and altered responses to endogenous growth factors as well as exogenous factors including therapeutic interventions seem to be dealt with in an ecological fashion as the offspring occupy niches.

But is the ecological model the correct model to use? How *de novo* tumors arise is not known. Our understanding of cancer suggests that *de novo* tumors arise in the same way as progressive tumors, just that the *de novo* tumor arises more rapidly. However, it is not clear how the many genetic mutations arise in the *de novo* tumors and how the tumors arise without passing through slower growing intermediate stages. In these situations of explosive growth, the whole manifests itself in a total that is greater than the sum of its parts. In other words, is ecological succession necessarily at play in these tumors or do the tumor cells escape true ecological selection via unknown, nonlinear mechanisms through which emergent glioblastoma behavior is manifest? In nonlinear systems, the end is exquisitely sensitive to initial conditions; subtle changes in starting conditions result in profoundly variable results. If this reflects reality in glioblastoma, what roles do the glioma stem cells play as tumor initiating cells?

In this regard, as anticipated by Ignatova and colleagues [17], the tumor cells have a limited repertoire of responses, limited by their overall driving genetic mutations [65] and altered epigenetic responses. However, within any cluster of cells (viewed here as a self organizing system), information flows between cells may be being nurtured by only a few glioma stem cells within their tumor-mesenchymal niche. In this system, the organotypic cell clusters are being guided not only by their own individually altered response repertoire to biological effectors (stimuli) but also by inanimate microenvironmental conditions (cues) such as access to nutrition, barriers to spread, and space to proliferate. The information supplied by the cues is subtle and can be overlooked making certain emergent properties appear to arise from mysterious origins [66]. Thus the cytotypic maps drawn by Burger and Kleihues [2] in which there is marked cytologic heterogeneity within the tumor, but relatively monotonous cellular profiles regionally, seem accessible via an understanding of microregional adaptation to both stimuli and cues. Here, we see necrosis as a reflection of failed adaptation and angiogenesis and migration as viable ones, albeit in a limited, host destructive sense. Although Darwinism may appear as the correct model, malignant progression and concomitant increased growth rates are only short-term positives as cells adapt to their micro-environments. Darwinism, strictly applied, is based on many generations of the host-agent interaction and is not the proper model.

In 2000, Hanahan and Weinberg [67] proposed six key requisites of a malignant cell: “self-sufficiency in growth signals, insensitivity to growth-inhibitory signals, evasion of programmed cell death, limitless replicative potential, sustained angiogenesis, and tissue invasion and metastasis.” The tumor stem cell concept addresses the majority of these requisites in surprisingly, but biologically economical, ways. They also hypothesized, with little more than histologic evidence to guide them, that “apparently normal bystanders such as fibroblasts and endothelial cells must play key roles in driving tumor cell proliferation.” Again, the tumor stem cell concept has directly revealed mechanisms whereby these observations are supported. The findings noted above related to glioma stem cells active in their niche provide a conceptual lens through which these cell to cell and cell to stroma information signals come into focus. There are many implications for present and future research.

Many recent experiments have strived to correlate cytologic structure and expression phenotypes with biological function. However, the histologic studies have been done on formalin-fixed, paraffin embedded tissues taken from regions, by definition, microscopically removed from the frozen section blocks from which the DNA and RNA were extracted. The data derived from glioma stem cell studies indicate that regional expression will be variable and dependent upon the micro-environment. Grinding up tissue from which to extract DNA and RNA will only provide an average answer related to the size of the tissue studied but will serve as a starting point. As data are collected from tumors relatively homogeneous with one histologic type, hypotheses can be generated to be answered by more precise methods. To address such concerns new techniques in microdissection, cell culture, and microenvironmental manipulation in xenografts will have to be developed in order to better understand the rich, organic interactions among glioma stem cells and their cellular and mesenchymal constituents.

To neuropathologists, glioblastoma has long been a tumor that represented a complex system of migrating pleomorphic tumor cells, proliferating blood vessels, infiltrating inflammatory cells, and necrosis. Now, the glioma stem cell concept brings these elements together into a collective whole interacting with microenvironmental influences in complex ways. The new paradigm of glioblastoma biology will be that genetic changes should be understood in a three-dimensional framework as they relate not only to the tumor cells themselves but also to the multicellular hierarchical unit, not isolated from, but responsive to, its local milieu. In this way we will come to better appreciate the impact our therapeutic interventions have on the regional phenotypic heterogeneity that exists within the tumor and the intercellular communications directing adaptation and progression.

## Figures and Tables

**Figure 1:**
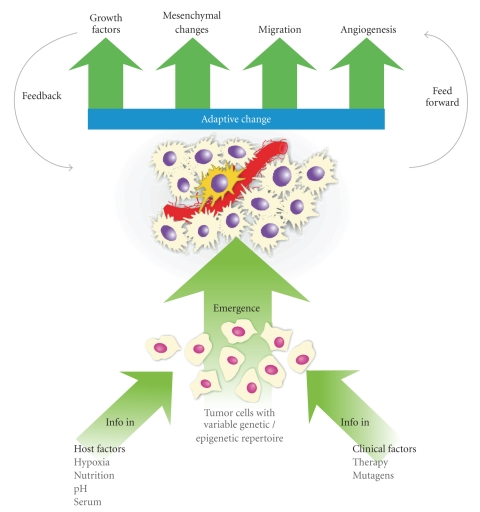
The concept of a self-organizing system with emergent properties as related to glioblastoma.

**Figure 2:**
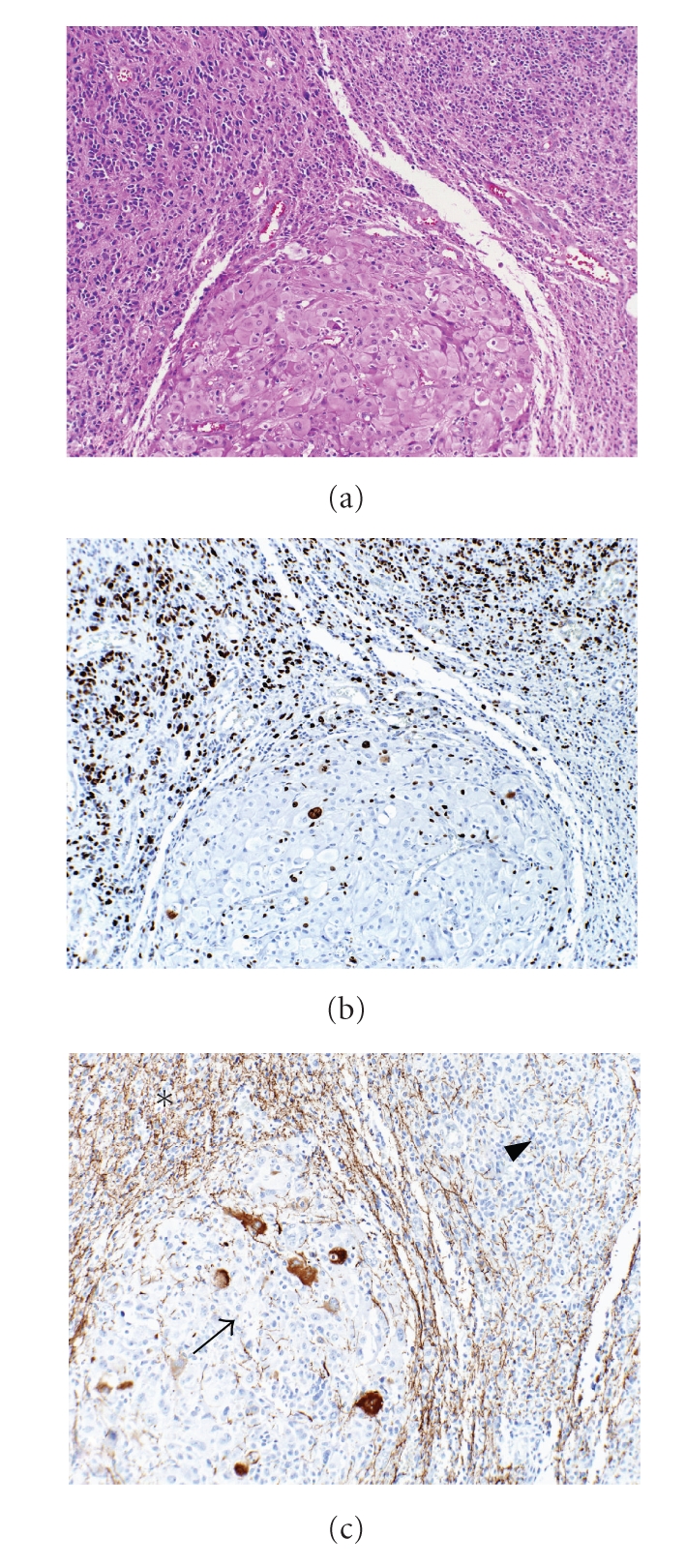
Microhomogeneity of a glioblastoma. (a) H&E stained section demonstrating two distinct cell morphologies with a nodule of large cells in the lower center surrounded by small cells in the upper portions of the photograph. (b) MIB-1 immunohistochemistry demonstrates the nodule of large cells in the lower center to have a low proliferation index relative to the small cells. (c) Neurofilament protein immunohistochemistry shows axons coursing through the small cells in the upper left region of the tumor (asterisk) with loss of axons in the upper right (arrowhead) and in the nodule of large cells (arrow).
